# Non-alcoholic fatty liver disease and cognitive function in middle-aged adults: the CARDIA study

**DOI:** 10.1186/s12876-021-01681-0

**Published:** 2021-03-02

**Authors:** Yariv Gerber, Lisa B. VanWagner, Kristine Yaffe, James G. Terry, Jamal S. Rana, Jared P. Reis, Stephen Sidney

**Affiliations:** 1 Department of Epidemiology and Preventive Medicine, School of Public Health, Sackler Faculty of Medicine, Tel Aviv University, 6997801 Tel Aviv, Israel; 2 Kaiser Permanente Northern California, Oakland, CA USA; 3 School of Public Health, University of California Berkeley, Berkeley, CA USA; 4 Northwestern University Feinberg School of Medicine, Chicago, IL USA; 5 Department of Medicine, University of California San Francisco, San Francisco, CA USA; 6 Vanderbilt University Medical Center, Nashville, TN USA; 7 National Heart Lung and Blood Institute, Bethesda, MD USA

**Keywords:** Non-alcoholic fatty liver disease, Cognitive performance, Cardiovascular disease, Neurological risk factors, Cognitive decline

## Abstract

**Background:**

Non-alcoholic fatty liver disease (NAFLD) is associated with cardiovascular disease (CVD) risk factors that have been linked to cognitive decline. Whether NAFLD is associated with cognitive performance in midlife remains uncertain.

**Methods:**

Coronary Artery Risk Development in Young Adults study participants with CT examination and cognitive assessment at Y25 (2010–2011; n = 2809) were included. Cognitive function was reassessed at Y30. NAFLD was defined according to liver attenuation and treated both continuously and categorically (using ≤ 40 and ≤ 51 Hounsfield units to define severity) after exclusion for other causes of liver fat. Cognitive tests including the Digit Symbol Substitution (processing speed), Rey Auditory Verbal Learning (verbal memory), and Stroop (executive function) were analyzed with standardized z-scores. Linear models were constructed to (a) examine the cross-sectional associations of NAFLD with cognitive scores and (b) evaluate its predictive role in 5-year change in cognitive performance.

**Results:**

Participants’ mean age (Y25) was 50.1 (SD 3.6) years (57% female; 48% black), with 392 (14%) having mild NAFLD and 281 (10%) having severe NAFLD. NAFLD was positively associated with CVD risk factors and inversely associated with cognitive scores. However, after adjustment for CVD risk factors, no associations were shown between NAFLD and cognitive scores (all βs ≈ 0). Similarly, no associations were observed with 5-year cognitive decline. CVD history, hypertension, smoking, diabetes and hypertriglyceridemia showed stronger associations with baseline cognitive scores and were predictive of subsequent cognitive decline (all *P* ≤ .05).

**Conclusion:**

Among middle-aged adults, inverse associations between NAFLD and cognitive scores were attenuated after adjustment for CVD risk factors, with the latter predictive of poorer cognitive performance both at baseline and follow-up.

## Introduction

Cognitive aging has been the focus of recent scientific interest, fueled by the rapid growth of the U.S. population age 65 and older [1–3]. While the process of cognitive aging takes place over decades [4], an increasing body of evidence suggests that early changes occur in midlife [5, 6]. Previous studies show that exposure to cardiovascular disease (CVD) risk factors in midlife is associated with an increased risk of dementia [7, 8]. Furthermore, in non-elderly individuals without dementia, classic CVD risk factors including hypertension, diabetes mellitus, smoking and obesity are predictive of cognitive performance [9–12], with both causal mechanisms and epiphenomenon having been proposed [13].

Non-alcoholic associated fatty liver disease (NAFLD), an accumulation of extra fat in liver cells that can lead to inflammation, liver fibrosis, cirrhosis and liver cancer, is an obesity-related condition that has reached an epidemic proportion [14, 15]. As recently reviewed, NAFLD is the most common cause of chronic liver disease worldwide, with a global prevalence of 25% and an enormous clinical and economic burden [16]. NAFLD often coexists with classic CVD risk factors [17, 18]. Whether NAFLD is associated with cognitive decline remains an important clinical question with potential implications for preventive interventions. Notably, the presence of cognitive deficits is common in patients diagnosed with other chronic liver diseases, such as primary biliary cholangitis [19], and might potentially appear in earlier, pre-cirrhotic stages of liver disease [20]. As such, NAFLD has been linked to increased risk of carotid atherosclerosis [21], a potential risk factor for cognitive impairment [22, 23], and was inversely associated with measures of early brain health [24, 25]. Specifically, population-based cross-sectional analyses have examined the relationship of NAFLD with cognitive performance in middle-aged adults, providing contradicting results [26, 27]. To date, however, prospective studies evaluating NAFLD in relation to change in cognitive function in midlife are not available. The Coronary Artery Risk Development in Young Adults (CARDIA) study is uniquely positioned to address this gap in knowledge with its diverse cohort and rigorous ascertainment of risk factors for cognitive aging repeatedly measured over time.

## Methods

### Study sample

CARDIA is a multicenter population-based cohort study of the development and determinants of CVD in black and white young adults recruited from 1985–1986 at 18–30 years of age across 4 U.S. cities (Birmingham, AL; Chicago, IL; Minneapolis, MN; and Oakland, CA). The study design has been described in detail elsewhere [28]. Nine examinations have been completed to date. Informed consent was obtained at each follow‐up examination and the study was approved by the Institutional Review Boards at each CARDIA site (University of Alabama Birmingham; Northwestern University; University of Minneapolis; Kaiser Permanente). The study protocol was in accordance to guidelines of the Institutional Review Boards. The present study includes participants who underwent both comprehensive cognitive function assessment [10] and computed tomography (CT) scanning of both the thorax and abdomen as part of the 25-year follow-up examination (Y25; 2010–2011) [29, 30]. Cognitive function was reassessed at the 30-year follow-up examination (Y30; 2015–2016) [31].

**Fig. 1:**
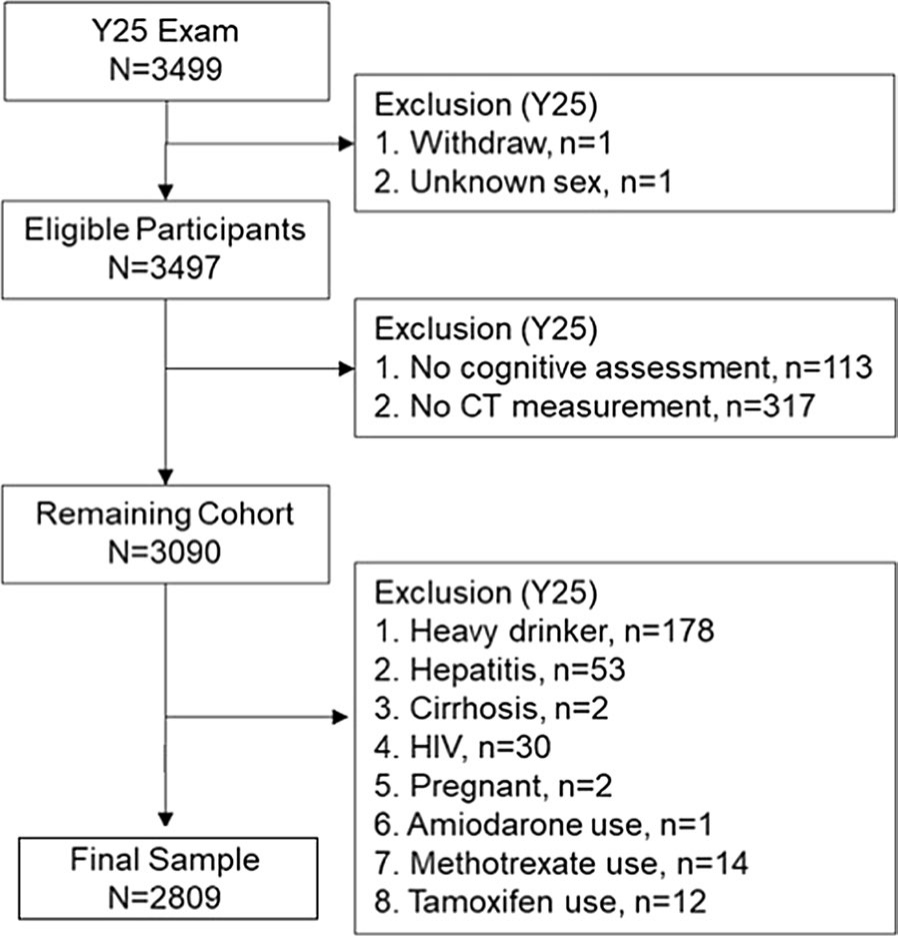
Study flow chart. Excessive alcohol consumption (heavy drinking) was defined as ≥ 20 g/day in women and ≥ 30 g/day in men. CT, computed tomography; HIV, human immunodeficiency virus

As described previously [29], there were 3499 participants (46% men, 51% black) who attended the Y25 examination. Participants were excluded from the CT exam if they weighed more than 450 lbs. (204 kg) or were unable to fit within the CT gantry. Also excluded were those without cognitive assessment, those missing measurements for liver fat, pregnant women, those with a self-reported history of hepatitis C or cirrhosis, and those with a risk factor for chronic liver disease (e.g., intravenous drug use) or with a potential cause of secondary hepatic steatosis: alcohol consumption ≥ 20 g/day in women and ≥ 30 g/day in men, self-reported human immunodeficiency virus (HIV), and medications known to cause hepatic steatosis. The remaining 2809 participants formed the sample population (Fig. 1). Of the sample population, 2369 had their cognitive function reassessed at the Y30 examination.

### Clinical measurements

Standardized protocols for data collection were used across study centers and measurements have previously been described [28], and are available online (https://www.cardia.dopm.uab.edu). Data of the Y25 follow-up examination were used. Demographics, alcohol consumption, smoking and other lifestyle habits were ascertained through questionnaires. Medication use was reported by participants who also brought in medications for verification. Personal CVD history was reported in a questionnaire and included myocardial infarction, angina pectoris, heart failure, valvular heart disease, peripheral vascular disease and stroke or transient ischemic attack. Cigarette smoking was defined as smoking at least 5 cigarettes per week almost every week. If answered “yes”, the subject was asked if he or she still smoked regularly, and those who responded “no” were considered to be past smokers. Measured height and weight were used to calculate body mass index (BMI) as weight in kilograms divided by height in meters squared; obesity was defined as BMI ≥ 30 kg/m^2^. Exam data on blood pressure measurements were used and hypertension was defined as systolic blood pressure ≥ 140 mm Hg, diastolic blood pressure ≥ 90 mm Hg or use of blood pressure lowering medications [32].

### Biochemical measurements

Participants were asked to fast for at least 12 h and to avoid smoking and heavy physical activity for at least 2 h before each examination. Blood was drawn, separated and plasma frozen to − 70 °C prior to analysis in a central laboratory. Diabetes was defined as fasting plasma glucose ≥ 126 mg/dL, oral glucose tolerance test ≥ 200 mg/dL, glycosylated hemoglobin ≥ 6.5% or use of glucose lowering medications. Hypercholesterolemia was defined as total cholesterol ≥ 240 mg/dL or use of lipid lowering medications. Hypertriglyceridemia was defined as total triglycerides ≥ 150 mg/dL.

### CT measures of liver attenuation and abdominal adipose tissues

The CT protocol included the heart and abdomen using a non-contrast CT scan performed using GE (GE 750HD 64 and GE LightSpeed VCT 64 Birmingham and Oakland Centers, respectively; GE Healthcare, Waukesha, Wisconsin) or Siemens (Sensation 64, Chicago and Minneapolis Centers; Siemens Medical Solutions, Erlangen, Germany) multidetector CT scanners and has been described previously [30]. Quality control and image analysis was performed at a core reading center (Wake Forest University Health Sciences, Winston-Salem, North Carolina). Measurement of liver attenuation (LA) was performed in the right lobe of the liver using CT slices through the upper abdomen and was reported as the average of 9 measurements on 3 slices using circular regions of interest of 2.6 cm^2^. The interclass correlation coefficient between different readers on a random selected sample of 156 participants was 0.98 for LA, indicating high reproducibility of CT-measured LA in this study [24].

LA was analyzed both as a continuous and categorical variable. Low levels of LA are equivalent to high levels of liver fat. For example, a liver-to-spleen ratio < 1.0 is comparable to using a LA cut-off of ≤ 51 Hounsfield Units (HU) for the diagnosis of mild liver fat [24]. A hepatic attenuation of ≤ 40 HU represents fatty change of approximately 30% and is more indicative of moderate-severe hepatic steatosis [30]. We therefore categorized LA into 3 groups: > 51 HU, no NAFLD; ≤ 51 HU and > 40 HU, mild NAFLD; and ≤ 40 HU, severe NAFLD. The methods for assessment of abdominal adiposity have also been described previously [30, 33]. Total abdomen adipose tissue (TAAT), subcutaneous adipose tissue (SAT), and visceral adipose tissue (VAT) were assessed.

### Cognitive function assessment

CARDIA technicians who underwent centralized formal training and certification administered a battery of 3 cognitive tests representing distinct domains of cognition at the Y25 and Y30 examinations. These included the Digit Symbol Substitution Test (DSST), the Rey Auditory Verbal Learning Test (RAVLT), and the Stroop Test. DSST, a subtest of the Wechsler Adult Intelligence Scale, assesses attention, working memory, psychomotor speed, and executive function, with higher scores indicating better performance, with a range of 0 to 133 [34]. RAVLT is a test of verbal memory. Scores on the delayed test were used, with higher scores indicating better performance, with a range of 0 to 15 [35, 36]. The Stroop Test of executive function uses 3 subtests. We calculated an interference score by subtracting the score on subtest II from subtest III, with a higher interference score indicating worse performance [37, 38]. For ease of interpretation, all cognitive test scores were transformed into standardized *z*-scores, with positive values indicating better performance and negative values indicating worse performance. A composite cognitive function score was computed by transforming each of the 3 tests to standardized *z*-scores and averaging the summed total [31]. Pearson's correlation coefficients between the standardized cognitive test scores at Y25 were as follows: DSST and RAVLT, r = 0.41; DSST and Stroop, r = 0.43; RAVLT and Stroop, r = 0.28. The corresponding correlations between the same tests measured at Y25 and Y30 were r = 0.83 for DSST; r = 0.70 for RAVLT; and r = 0.70 for Stroop.

### Statistical analysis

Analyses were performed using SAS 9.4 (SAS institute, Cary, NC) and IBM SPSS Statistics, version 25 (IBM SPSS Inc.). Characteristics across NAFLD categories (LA > 51 HU for no NAFLD, 40 < LA ≤ 51 HU for mild NAFLD, and LA ≤ 40 HU for severe NAFLD) are presented as mean (SD) or median (IQR) for continuous variables and as frequencies for categorical variables, and were compared by analysis of variance (ANOVA), Kruskal Wallis, or chi-square tests, as appropriate.

Multivariable linear regression models were constructed to assess the cross-sectional associations (beta coefficients and standard errors) of NAFLD, other CT-measured fat indices, and classic CVD risk factors with cognitive function test scores as measured at the Y25 exam. NAFLD was modeled either as a categorical variable (as specified above) or a continuous variable (LA). Because the distribution of the latter was negatively skewed, a square transformation was performed. As mentioned, all cognitive test scores (DSST, RAVLT, and Stroop) were transformed into standardized z-scores, with positive values indicating better performance and negative values indicating worse performance. A composite cognitive function score was additionally computed by averaging test-specific standardized z-scores of the three cognitive measures. For each cognitive test, we used a sequential adjustment approach controlling for sociodemographic variables (age, race, sex, study center, and education) (model 1) as well as CVD risk factors (CVD history, hypertension, diabetes mellitus, smoking, hypercholesterolemia, hypertriglyceridemia, and obesity) (model 2).

The predictive role of NAFLD, other CT-measured fat indices, and classic CVD risk factors measured at Y25 in cognitive test scores obtained at Y30 was evaluated using multivariable linear regression models as described above, with the exception that the baseline cognitive score for each test was added as a covariate to the respective models. Effect modification of the associations between LA and cognitive scores by age, sex, and race was assessed by testing 2-way interaction terms in the above-specified models. About 16% of Y25 participants did not take part at the Y30 cognitive assessment. To account partly for this potential bias, the probability of Y25 participants to attend the Y30 examination was estimated using multivariable logistic regression. Inverse probability weights (IPW) were then applied [39] and the weighted and non-weighted analyses compared. Missing values of any of the covariates used in the analysis did not exceed 1%. A two-sided *P* value ≤ 0.05 was considered statistically significant.

## Results

At study baseline (Y25), the mean (SD) age of the participants (n = 2809) was 50.1 (3.6) years, 48% were black, 43% were male, and the mean (SD) years of education was 15.1 (2.7). Classic CVD risk factors were prevalent in this cohort, including 45% with obesity, 37% current or past smokers, 35% with hypertension, 24% with hypercholesterolemia, and 14% with diabetes (Table 1). According to CT-measured LA, 76% had no NAFLD, 14% had mild NAFLD, and 10% had severe NAFLD. NAFLD was overrepresented among males and was generally associated with a worse cardiovascular profile. Specifically, participants with mild or severe NAFLD had higher prevalence of hypertension, diabetes, hypercholesterolemia, hypertriglyceridemia and obesity, compared with their NAFLD-free counterparts. Also, participants with NAFLD exhibited higher levels of CT fat measures including VAT volume. Finally, NAFLD was inversely associated with DSST, RAVLT and Stroop scores at baseline, although not in a clear dose–response fashion (Table 1).

**Table 1:** Baseline characteristics, overall and by NAFLD category, among year 25 CARDIA participants

Variable	Overall (n = 2809)	Liver attenuation			*P*
		No NAFLD	Mild NAFLD	Severe NAFLD	
		> 51 HU (n = 2136)	> 40–51 HU (n = 392)	≤ 40 HU (n = 281)	
Liver attenuation, HU, median (IQR)	57.7 (51.5–62.3)	59.9 (56.3–64.0)	47.3 (44.3–49.4)	31.3 (23.6–36.8)	< .001^a^
*Socio-demographics*					
Age, year, mean ± SD	50.1 ± 3.6	50.0 ± 3.7	50.3 ± 3.6	50.5 ± 3.6	.034
Male, n (%)	1198 (43)	823 (39)	219 (56)	156 (56)	< .001
Black, n (%)	1344 (48)	1038 (49)	190 (49)	116 (41)	.067
Education, year, mean ± SD	15.1 ± 2.7	15.1 ± 2.7	14.8 ± 2.6	15.0 ± 2.7	.040
*CVD risk factors*					
Personal CVD history, n (%)	402 (14)	303 (14)	55 (14)	44 (16)	.79
Hypertension, n (%)	971 (35)	632 (30)	175 (45)	164 (58)	< .001
Smoking, n (%)					.010
Never	1749 (62)	1363 (65)	219 (56)	167 (60)	
Past	607 (22)	435 (21)	99 (25)	73 (26)	
Current	420 (15)	311 (15)	71 (18)	38 (14)	
Diabetes mellitus, n (%)	391 (14)	187 (9)	82 (21)	122 (43)	< .001
Hypercholesterolemia, n (%)	668 (24)	449 (21)	122 (31)	97 (35)	< .001
Hypertriglyceridemia, n (%)	545 (20)	282 (13)	132 (34)	131 (47)	< 0.001
Obesity, n (%)	1257 (45)	746 (35)	285 (73)	226 (80)	< .001
*CT fat measures*					
TAAT, cm^3^, mean ± SD	489 ± 217	442 ± 201	613 ± 194	678 ± 191	< .001
SAT, cm^3^, mean ± SD	340 ± 170	314 ± 165	413 ± 161	430 ± 154	< .001
VAT, cm^3^, mean ± SD	131 ± 73	111 ± 59	176 ± 69	222 ± 83	< .001
*Cognitive test scores*					
DSST, symbols, mean ± SD	70.0 ± 16.0	70.6 ± 16.3	67.6 ± 15.6	68.9 ± 14.4	.002
RAVLT, words, mean ± SD	8.3 ± 3.3	8.4 ± 3.3	7.9 ± 3.1	8.0 ± 3.1	.003
Stroop, seconds plus errors, mean ± SD	22.9 ± 10.8	22.7 ± 10.7	24.3 ± 11.6	22.9 ± 10.2	.028

CVD, cardiovascular disease; DSST, digit symbol substitution test; HU, Hounsfield Unit; NAFLD, metabolic associated fatty liver disease; RAVLT, Rey auditory verbal learning test; SAT, subcutaneous adipose tissue; SD, standard deviation; TAAT, total abdomen adipose tissue; VAT, visceral adipose tissue

^a^Kruskal Wallis Test

The cross-sectional associations between LA and standardized cognitive test scores are presented in Table 2. Adjusted for sociodemographic measures, each 1 SD lower LA (1 SD = 12 HU; low LA = high fatty liver) was significantly but weakly associated with lower scores in the Stroop and the composite cognitive outcome. The associations with DSST and RAVLT were weaker and nonsignificant. Further adjustment for classic CVD risk factors nearly nullified the associations of LA with all cognitive test scores. Using categories instead of the continuous LA variable resulted in weak and nonsignificant associations with cognitive scores, except one between mild NAFLD and Stroop in the sociodemographic-adjusted model, which was also attenuated with further adjustment for CVD risk factors. In contrast, CVD history (RAVLT, composite), hypertension (DSST, RAVLT, Stroop, composite), smoking (DSST, RAVLT, Stroop, composite), diabetes (DSST, Stroop, composite), hypercholesterolemia (RAVLT, Stroop, composite) and hypertriglyceridemia (DSST, Stroop, composite) were all significantly associated with lower cognitive scores in the sociodemographic-adjusted model. Many of these associations remained significant in the fully adjusted model, including CVD history (RAVLT, composite), hypertension (DSST, RAVLT, Stroop, composite), smoking (DSST, RAVLT, Stroop, composite), hypercholesterolemia (RAVLT) and hypertriglyceridemia (DSST). Neither obesity nor CT-measured fat variables showed any relationship with cognitive scores, except for an association of TAAT and VAT with DSST, which was attenuated with adjustment for CVD risk factors (Table 2). No clinically meaningful interactions were shown between LA and age, race, or sex in the adjusted models.

**Table 2:** Adjusted cross-sectional differences in standardized cognitive scores associated with NAFLD, other CT fat measures, and classic CVD risk factors at year 25 among CARDIA participants

Variable	Model 1				Model 2			
	DSST	RAVLT	Stroop test	Composite score	DSST	RAVLT	Stroop	Composite score
*CT fat measures*								
LA, 1 SD lower (12 HU)	− .03 (.02)	− .01 (.02)	− .04 (.02)*	− .03 (.01)*	− .01 (.02)	.01 (.02)	− .01 (.02)	.00 (.01)
TAAT, 1 SD higher (217 cm^3^)	− .04 (.02)*	.01 (.02)	− .03 (.02)	− .02 (.01)	− .03 (.02)	.03 (.03)	.02 (.03)	.01 (.02)
SAT, 1 SD higher (170 cm^3^)	− .03 (.02)	.02 (.02)	− .03 (.02)	− .01 (.01)	− .03 (.03)	.04 (.03)	.00 (.03)	.00 (.02)
VAT, 1 SD higher (73 cm^3^)	− .04 (.02)*	− .01 (.02)	− .03 (.02)	− .02 (.01)	.00 (.02)	.01 (.02)	.03 (.02)	.01 (.02)
*CVD risk factors*								
CVD history	− .09 (.05)	− .14 (.05)**	− .09 (.05)	− .10 (.03)**	− .07 (.05)	− .12 (.05)*	− .07 (.05)	− .08 (.03)*
Hypertension	− .12 (.04)**	− .14 (.04)**	− .16 (.04)**	− .14 (.03)**	− .08 (.04)*	− .12 (.04)**	− .12 (.04)**	− .10 (.03)**
Current smoking	− .36 (.05)**	− .24 (.05)**	− .16 (.05)**	− .25 (.03)**	− .35 (.05)**	− .23 (.05)**	− .16 (.05)**	− .24 (.03)**
Diabetes Mellitus	− .12 (.05)*	− .05 (.05)	− .14 (.05)**	− .10 (.03)**	− .07 (.05)	.02 (.05)	− .05 (.05)	− .04 (.04)
Hypercholesterolemia	− .02 (.04)	− .12 (.04)**	− .08 (.04)*	− .08 (.03)**	.03 (.04)	− .10 (.04)*	− .04 (.04)	− .04 (.03)
Hypertriglyceridemia	− .13 (.04)**	− .04 (.04)	− .12 (.04)**	− .09 (.03)**	− .09 (.04)*	.00 (.04)	− .07 (.05)	− .05 (.03)
Obesity	− .05 (.03)	.01 (.04)	− .06 (.04)	− .03 (.02)	− .03 (.04)	.03 (.04)	− .04 (.04)	− .01 (.03)
NAFLD categories^a^								
None (LA ≥ 51 HU)	0 (ref.)	0 (ref.)	0 (ref.)	0 (ref.)	0 (ref.)	0 (ref.)	0 (ref.)	0 (ref.)
Mild (40 < LA < 51 HU)	− .05 (.05)	.02 (.05)	− .11 (.05)*	− .05 (.03)	.01 (.05)	.04 (.05)	− .05 (.05)	.00 (.04)
Severe (LA ≤ 40 HU)	− .04 (.05)	− .05 (.06)	− .06 (.06)	− .06 (.04)	.06 (.06)	.00 (.06)	.03 (.07)	.02 (.04)

Values represent β (SE); negative coefficients indicate inferior cognitive performance

Model 1: Adjusted for study center, age, race, sex, and education

Model 2: Further adjusted for CVD risk factors (CVD history, hypertension, smoking, diabetes mellitus, hypercholesterolemia, hypertriglyceridemia, and obesity)

CVD, cardiovascular disease; DSST, digit symbol substitution test; LA, liver attenuation; NAFLD, metabolic associated fatty liver disease; RAVLT, Rey auditory verbal learning test; SAT, subcutaneous adipose tissue; SD, standard deviation; TAAT, total abdomen adipose tissue; VAT, visceral adipose tissue. DSST: n = 2793; RAVLT: n = 2786; Stroop: n = 2773; composite score: n = 2755

*P ≤ .05; **P ≤ .01

^a^Modeled separately from liver attenuation as an alternative definition for NAFLD

The predictive role of LA as well as other covariates in change in cognitive scores between the Y25 and Y30 examinations is shown in Table 3. After adjustment for baseline cognitive test scores and sociodemographic variables, LA, either as a continuous or a categorical variable, was not associated with changes in standardized cognitive scores. In multivariable models further adjusted for CVD risk factors, severe NAFLD was associated with marginally better Stroop and composite scores. TAAT, SAT, and VAT were significantly associated with DSST deterioration in models adjusted for baseline DSST and sociodemographic variables. However, the associations were attenuated upon further adjustment for CVD risk factors. No associations were observed between CT fat indices and RAVLT, Stroop, or the composite cognitive score. In contrast, most classic CVD risk factors were significantly associated with cognitive score deterioration. In models adjusted for baseline cognitive test scores and sociodemographic variables, CVD history (RAVLT, Stroop, composite), hypertension (DSST, RAVLT, composite), smoking (RAVLT, Stroop, composite), diabetes (DSST, RAVLT, composite), hypertriglyceridemia (DSST, RAVLT, Stroop, composite) and obesity (DSST) were associated with poorer cognitive performance at follow-up. Many of the associations including CHD history (RAVLT, Stroop, composite), hypertension (DSST), smoking (RAVLT, Stroop, composite), diabetes (composite) and hypertriglyceridemia (RAVLT, Stroop, composite) remained significant in the fully adjusted models (Table 3).

**Table 3:** Adjusted prospective changes in standardized cognitive scores at Y30 associated with NAFLD, other CT fat measures, and classic CVD risk factors measured at Y25 among CARDIA participants

Variable	Model 1				Model 2			
	DSST	RAVLT	Stroop test	Composite score	DSST	RAVLT	Stroop	Composite score
*CT fat measures*								
LA, 1 SD lower (12 HU)	− .02 (.01)	− .02 (.02)	.01 (.02)	− .01 (.01)	.01 (.01)	.00 (.02)	.02 (.02)	.01 (.01)
TAAT, 1 SD higher (213 cm^3^)	− .04 (.01)**	− .01 (.01)	.01 (.02)	− .01 (.01)	− .01 (.02)	− .02 (.02)	.01 (.02)	− .01 (.01)
SAT, 1 SD higher (166 cm^3^)	− .03 (.01)**	− .01 (.02)	.01 (.02)	− .01 (.01)	− .02 (.02)	− .03 (.02)	− .01 (.02)	− .02 (.01)
VAT, 1 SD higher (73 cm^3^)	− .03 (.01)**	− .01 (.01)	.01 (.02)	− .01 (.01)	.00 (.02)	.00 (.02)	.02 (.02)	.01 (.01)
*CVD risk factors*								
CVD history	− .03 (.03)	− .13 (.04)**	− .10 (.04)*	− .06 (.02)**	− .02 (.03)	− .12 (.04)**	− .10 (.04)*	− .06 (.02)*
Hypertension	− .09 (.02)**	− .08 (.03)*	− .02 (.03)	− .05 (.02)**	− .06 (.03)*	− .05 (.03)	.00 (.04)	− .02 (.02)
Current smoking	− .06 (.03)	− .10 (.04)*	− .14 (.05)**	− .08 (.03)**	− .06 (.03)	− .09 (.04)*	− .13 (.05)**	− .07 (.03)**
Diabetes Mellitus	− .10 (.03)**	− .12 (.04)**	− .01 (.04)	− .08 (.03)**	− .06 (.04)	− .09 (.05)	.00 (.05)	.06 (.03)*
Hypercholesterolemia	− .04 (.03)	− .06 (.03)	− .02 (.04)	− .02 (.02)	− .01 (.03)	− .02 (.04)	.00 (.04)	.01 (.02)
Hypertriglyceridemia	− .07 (.03)**	− .10 (.04)**	− .08 (.04)*	− .08 (.02)**	− .04 (.03)	− .08 (.04)*	− .09 (.04)*	− .07 (.02)**
Obesity	− .07 (.02)**	.00 (.03)	.03 (.03)	− .01 (.02)	− .04 (.02)	.04 (.03)	.03 (.03)	.01 (.02)
NAFLD categories^a^								
None (LA ≥ 51 HU)	0 (ref.)	0 (ref.)	0 (ref.)	0 (ref.)	0 (ref.)	0 (ref.)	0 (ref.)	0 (ref.)
Mild (40 < LA < 51 HU)	− .02 (.03)	− .03 (.04)	− .05 (.05)	− .03 (.03)	.02 (.04)	.00 (.05)	− .04 (.05)	.00 (.03)
Severe (LA ≤ 40 HU)	− .04 (.04)	− .02 (.05)	.09 (.05)	.01 (.03)	.04 (.04)	.05 (.05)	.12 (.06)*	.08 (.03)*

Values represent β (SE); negative coefficients indicate greater cognitive decline

Model 1: Adjusted for baseline (Y25) cognitive score (of each respective test), study center, age, race, sex, and education

Model 2: Further adjusted for CVD risk factors (CVD history, hypertension, smoking, diabetes mellitus, hypercholesterolemia, hypertriglyceridemia, and obesity)

CVD, cardiovascular disease; DSST, digit symbol substitution test; LA, liver attenuation; NAFLD, metabolic associated fatty liver disease; RAVLT, Rey auditory verbal learning test; SAT, subcutaneous adipose tissue; SD, standard deviation; TAAT, total abdomen adipose tissue; VAT, visceral adipose tissue. DSST: n = 2355; RAVLT: n = 2366; Stroop: n = 2314; composite score: n = 2303

*P ≤ .05; **P ≤ .01

^a^Modeled separately from liver attenuation as an alternative definition for NAFLD

Approximately 16% of the participants included in the Y25 cross-sectional analysis did not attend the Y30 cognitive assessment. No significant differences between participants and nonparticipants were found in age and sex. However, nonparticipants were more likely to be black, less educated, and with worse CVD risk factor profile. They also obtained lower scores in all baseline cognitive tests compared with Y30 participants (all *P* < 0.01). Applying IPW to partially account for loss to follow-up between the Y25 and Y30 exams affected the results only minimally. For example, in the fully adjusted weighted model for change in the composite cognitive score, the β ± SE associated with a 1 SD lower LA was 0.01 ± 0.01 (*P* = 0.52), thus supporting the results of the non-weighted analysis.

## Discussion

In a large population-based epidemiological study of black and white middle-aged adults from 4 U.S. cities, the presence of NAFLD on CT was associated with less favorable cardiovascular risk profile and lower cognitive performance. The latter was based on 3 standardized tests evaluating different domains: DSST (processing speed), RAVLT (verbal memory), and Stroop (executive function). However, the crude, cross-sectional associations between NAFLD and all cognitive tests were attenuated after adjustment for sociodemographic and CVD risk factors. Moreover, NAFLD was not associated with subsequent decline in cognitive scores as assessed at the Y30 follow-up exam using the same battery of tests. Similarly, other CT fat indices including VAT showed little associations with cognitive performance both at baseline and at follow-up.

In a previous cross-sectional study, Seo et al. [26] analyzed data of 4472 subjects (mean age, 37 years) from the Third National Health and Nutritional Examination Survey (NHANES III), 874 of whom were classified as NAFLD by ultrasound. Subjects underwent 3 computer-administered tests to assess their cognitive function, including DSST, the Serial Digit Learning Test (learning, recall, and concentration), and the Simple Reaction Time Test (visual-motor speed). Adjusted for sociodemographic measures, participants with NAFLD exhibited lower performance in all cognitive tests. However, further adjustment for CVD risk factors attenuated the associations, but that of the Serial Digit Learning Test remained statistically significant. The authors speculated that NAFLD might affect brain function via either insulin resistance or inflammatory processes. As recently reviewed [40], liver and brain illnesses share common metabolic risk determinants, including insulin resistance, high blood pressure, overweight, sedentary lifestyle and hyperlipidemia. These variables frequently coexist with NAFLD and have been associated with enhanced cerebral small vessel disease, resulting in white matter lesions, cerebral microhemorrhages, and brain atrophy. In addition, NAFLD is characterized by increased inflammation which induces platelet activity, pro-coagulant imbalance and endothelial dysfunction, which may lead to cerebral vessel and microvascular changes [41]. Brain circulation might also be influenced, possibly damaging the cerebral blood flow and supply, which might eventually lead to microvascular ischemia, brain tissue damage, atrophy, and cognitive decline [40]. Most recently, Weinstein et al. [27] assessed the cross-sectional association between NAFLD and cognitive function among 1287 Framingham Heart Study 2nd and 3rd generation participants (mean age, 61 years). Abdomen CT was used to assess NAFLD and a cognitive battery testing memory, reasoning, visual perception, attention and executive function was administered. NAFLD was not associated with any of the cognitive tests.

Similar to the above-mentioned Framingham study, we did not observe an independent association between NAFLD and cognitive function at baseline. Specifically, adjustment for CVD risk factors practically nullified the already weak NAFLD-cognitive function association. In addition, NAFLD in our study was not predictive of cognitive function at follow-up. It is thus possible that the association of NAFLD with cognitive performance suggested previously is an epiphenomenon. For example, insulin resistance may partly account for the association, as it might play a role in both NAFLD pathogenesis and Alzheimer’s disease development [26, 42]. Several methodological aspects need to be considered. Misclassification of outcomes may have biased the results to the null. Although we examined different domains of cognitive function, performance on neuropsychological tests does not necessarily accurately reflect biological functioning and capacity of the brain [43]. A more thorough neuropsychological battery could have enhanced validity and reliability. While memory, executive function and processing speed were tested in the present study, other cognitive domains could have yielded different results. Some exposure misclassification is also likely. CT is a relatively insensitive measure of hepatic fat compared with hepatic triglyceride content measured by proton magnetic resonance spectroscope (MR spectroscopy) or MR proton density fat fraction (MR PDFF) [44]. Liver biopsy, the gold standard for diagnosis of NAFLD [45], is not feasible in epidemiologic studies given the risks associated with the procedure. NAFLD prevalence in CARDIA is on the lower end of the reported spectrum of disease and apart from assessing degree of hepatic fat, we are unable to assess for other markers of NAFLD severity, such as hepatic inflammation or fibrosis due to lack of contemporaneous measures of liver chemistries at the time of the CT examination in CARDIA [24]. Cognitive decline is already evident in middle age [5, 6], yet a slower decline between ages 50 and 65 years relative to older ages has been suggested in some cognitive domains [46]. For example, in the Whitehall II prospective cohort study, among men aged 45–49, 10 year decline in reasoning was − 3.6% while in those aged 65–70 it was − 9.6% [5]. Accordingly, changes in cognitive scores between the 2 CARDIA assessments performed 5 years apart might have been relatively small and difficult to detect considering the participants’ age (mean ± SD 50.1 ± 3.6 years at Y25 baseline). Furthermore, CARDIA participants who completed the Y30 visit were inherently healthier than nonparticipants. This healthy-participant effect likely resulted in an underestimation of decline rates. We attempted to address this methodological challenge by applying inverse probability of participation weights, which affected the results very minimally.

In contrast to NAFLD and other CT-measured fat indices, CVD and its major risk factors were significantly and independently associated with cognitive performance in our study. This relationship concerning middle-aged adults supports previous findings from CARDIA [10] and other settings [47, 48]. Our analysis also supports a recent CARDIA investigation that linked CVD risk factors to accelerated cognitive decline [12]. To this end, CVD history, smoking, hypertension, diabetes, and hypertriglyceridemia were all predictive of unfavorable changes in one or more of the cognitive domains during a 5-year follow-up. These findings, though not proving causality, call for improvements in clinical management of CVD and its major risk factors over the life course. Clinicians and public health professionals should act as advocates for improving cardiovascular health with the goal to slow down cognitive aging.

## Data Availability

The data and materials are available from CARDIA and can be obtained from Dr. Steve Sidney (steve.sidney@kp.org).

## References

[1] Frey W (2010). Baby boomers and the new demographics of America's seniors. Generations.

[2] Sidney S, Go AS, Jaffe MG, Solomon MD, Ambrosy AP, Rana JS (2019). Association between aging of the US population and heart disease mortality From 2011 to 2017. JAMA Cardiol.

[3] Plassman BL, Langa KM, Fisher GG, Heeringa SG, Weir DR, Ofstedal MB, Burke JR, Hurd MD, Potter GG, Rodgers WL, Steffens DC, Willis RJ, Wallace RB (2007). Prevalence of dementia in the United States: the aging, demographics, and memory study. Neuroepidemiology.

[4] Jack CR, Knopman DS, Jagust WJ, Shaw LM, Aisen PS, Weiner MW, Petersen RC, Trojanowski JQ (2010). Hypothetical model of dynamic biomarkers of the Alzheimer's pathological cascade. Lancet Neurol.

[5] Singh-Manoux A, Kivimaki M, Glymour MM, Elbaz A, Berr C, Ebmeier KP, Ferrie JE, Dugravot A (2012). Timing of onset of cognitive decline: results from Whitehall II prospective cohort study. BMJ.

[6] Salthouse TA (2016). Continuity of cognitive change across adulthood. Psychon Bull Rev.

[7] Kivipelto M, Ngandu T, Laatikainen T, Winblad B, Soininen H, Tuomilehto J (2006). Risk score for the prediction of dementia risk in 20 years among middle aged people: a longitudinal, population-based study. Lancet Neurol.

[8] Alonso A, Mosley TH, Gottesman RF, Catellier D, Sharrett AR, Coresh J (2009). Risk of dementia hospitalisation associated with cardiovascular risk factors in midlife and older age: the Atherosclerosis Risk in Communities (ARIC) study. J Neurol Neurosurg Psychiatry.

[9] Reijmer YD, van den Berg E, Dekker JM, Nijpels G, Stehouwer CD, Kappelle LJ, Biessels GJ (2012). Development of vascular risk factors over 15 years in relation to cognition: the Hoorn Study. J Am Geriatr Soc.

[10] Yaffe K, Vittinghoff E, Pletcher MJ, Hoang TD, Launer LJ, Whitmer R, Coker LH, Sidney S (2014). Early adult to midlife cardiovascular risk factors and cognitive function. Circulation.

[11] Debette S, Seshadri S, Beiser A, Au R, Himali JJ, Palumbo C, Wolf PA, DeCarli C (2011). Midlife vascular risk factor exposure accelerates structural brain aging and cognitive decline. Neurology.

[12] Yaffe K, Bahorik AL, Hoang TD, Forrester S, Jacobs DR, Lewis C, Lloyd-Jones D, Sidney S, Reis J (2020). Cardiovascular risk factors and accelerated cognitive decline in mid-life: the CARDIA study. Neurology..

[13] Jefferson AL (2014). Vascular risk factors and midlife cognition: rethinking the exposure window. Circulation.

[14] Loomba R, Sanyal AJ (2013). The global NAFLD epidemic. Nat Rev Gastroenterol Hepatol.

[15] Vernon G, Baranova A, Younossi ZM (2011). Systematic review: the epidemiology and natural history of non-alcoholic fatty liver disease and non-alcoholic steatohepatitis in adults. Aliment Pharmacol Ther.

[16] Younossi ZM, Koenig AB, Abdelatif D, Fazel Y, Henry L, Wymer M (2016). Global epidemiology of nonalcoholic fatty liver disease-Meta-analytic assessment of prevalence, incidence, and outcomes. Hepatology.

[17] Pacana T, Fuchs M (2012). The cardiovascular link to nonalcoholic fatty liver disease: a critical analysis. Clin Liver Dis.

[18] Stepanova M, Younossi ZM (2012). Independent association between nonalcoholic fatty liver disease and cardiovascular disease in the US population. Clin Gastroenterol Hepatol.

[19] Newton JL, Hollingsworth KG, Taylor R, El-Sharkawy AM, Khan ZU, Pearce R, Sutcliffe K, Okonkwo O, Davidson A, Burt J, Blamire AM, Jones D (2008). Cognitive impairment in primary biliary cirrhosis: symptom impact and potential etiology. Hepatology.

[20] Yilmaz Y, Ozdogan O (2009). Liver disease as a risk factor for cognitive decline and dementia: an under-recognized issue. Hepatology.

[21] Brea A, Mosquera D, Martin E, Arizti A, Cordero JL, Ros E (2005). Nonalcoholic fatty liver disease is associated with carotid atherosclerosis: a case-control study. Arterioscler Thromb Vasc Biol.

[22] Spence JD (2004). Carotid intima-media thickness and cognitive decline: what does it mean for prevention of dementia?. J Neurol Sci.

[23] Zhong W, Cruickshanks KJ, Huang G-H, Klein BEK, Klein R, Nieto FJ, Pankow JS, Schubert CR (2011). Carotid atherosclerosis and cognitive function in midlife: the Beaver Dam Offspring Study. Atherosclerosis.

[24] VanWagner LB, Terry JG, Chow LS, Alman AC, Kang H, Ingram KH, Shay C, Lewis CE, Bryan RN, Launer LJ, Jeffrey CJ (2017). Nonalcoholic fatty liver disease and measures of early brain health in middle-aged adults: The CARDIA study. Obesity (Silver Spring).

[25] Weinstein G, Zelber-Sagi S, Preis SR, Beiser AS, DeCarli C, Speliotes EK, Satizabal CL, Vasan RS, Seshadri S (2018). Association of nonalcoholic fatty liver disease with lower brain volume in healthy middle-aged adults in the Framingham study. JAMA Neurol.

[26] Seo SW, Gottesman RF, Clark JM, Hernaez R, Chang Y, Kim C, Ha KH, Guallar E, Lazo M (2016). Nonalcoholic fatty liver disease is associated with cognitive function in adults. Neurology.

[27] Weinstein G, Davis-Plourde K, Himali JJ, Zelber-Sagi S, Beiser AS, Seshadri S (2019). Non-alcoholic fatty liver disease, liver fibrosis score and cognitive function in middle-aged adults: the Framingham Study. Liver Int.

[28] Friedman GD, Cutter GR, Donahue RP, Hughes GH, Hulley SB, Jacobs DR, Liu K, Savage PJ (1988). CARDIA: study design, recruitment, and some characteristics of the examined subjects. J Clin Epidemiol.

[29] VanWagner LB, Wilcox JE, Colangelo LA, Lloyd-Jones DM, Carr JJ, Lima JA, Lewis CE, Rinella ME, Shah SJ (2015). Association of nonalcoholic fatty liver disease with subclinical myocardial remodeling and dysfunction: a population-based study. Hepatology.

[30] VanWagner LB, Ning H, Lewis CE, Shay CM, Wilkins J, Carr JJ, Terry JG, Lloyd-Jones DM, Jacobs DR, Carnethon MR (2014). Associations between nonalcoholic fatty liver disease and subclinical atherosclerosis in middle-aged adults: the Coronary Artery Risk Development in Young Adults Study. Atherosclerosis.

[31] McEvoy CT, Hoang T, Sidney S, Steffen LM, Jacobs DR, Shikany JM, Wilkins JT, Yaffe K (2019). Dietary patterns during adulthood and cognitive performance in midlife: The CARDIA study. Neurology.

[32] Gerber Y, Rana JS, Jacobs DR, Jr., Yano Y, Levine DA, Nguyen-Huynh MN, Lima JA, Reis JP, Zhao L, Liu K, Lewis CE, Sidney S. Blood pressure levels in young adulthood and midlife stroke incidence in a diverse cohort. Hypertension. 2021 (in press). 10.1161/HYPERTENSIONAHA.120.16535 PMC8035276 33775116

[33] Terry JG, Shay CM, Schreiner PJ, Jacobs DR, Sanchez OA, Reis JP, Goff DC, Gidding SS, Steffen LM, Carr JJ (2017). Intermuscular adipose tissue and subclinical coronary artery calcification in midlife: the CARDIA Study (Coronary Artery Risk Development in Young Adults). Arterioscler Thromb Vasc Biol.

[34] Wechsler D (1997). Wechsler Adult Intelligence Scale-III (WAIS-III).

[35] Rosenberg SJ, Ryan JJ, Prifitera A (1984). Rey Auditory-Verbal Learning Test performance of patients with and without memory impairment. J Clin Psychol.

[36] Schmidt M. Rey auditory verbal learning test: a handbook. Western Psychological Services Los Angeles (1996).

[37] Stroop JR (1935). Studies of interference in serial verbal reactions. J Exp Psychol.

[38] MacLeod CM (1991). Half a century of research on the Stroop effect: an integrative review. Psychol Bull.

[39] Robins JM, Hernán MA, Brumback B (2000). Marginal structural models and causal inference in epidemiology. Epidemiology.

[40] Lombardi R, Fargion S, Fracanzani AL (2019). Brain involvement in non-alcoholic fatty liver disease (NAFLD): a systematic review. Dig Liver Dis.

[41] Targher G, Day CP, Bonora E (2010). Risk of cardiovascular disease in patients with nonalcoholic fatty liver disease. N Engl J Med.

[42] Estrada LD, Ahumada P, Cabrera D, Arab JP (2019). Liver Dysfunction as a novel player in Alzheimer's progression: looking outside the brain. Frontiers in aging neuroscience.

[43] Weuve J, Proust-Lima C, Power MC, Gross AL, Hofer SM, Thiébaut R, Chêne G, Glymour MM, Dufouil C (2015). Guidelines for reporting methodological challenges and evaluating potential bias in dementia research. Alzheimers Dement.

[44] Cleveland E, Bandy A, VanWagner LB (2018). Diagnostic challenges of nonalcoholic fatty liver disease/nonalcoholic steatohepatitis. Clin Liver Dis (Hoboken).

[45] Chalasani N, Younossi Z, Lavine JE, Diehl AM, Brunt EM, Cusi K, Charlton M, Sanyal AJ (2012). The diagnosis and management of non-alcoholic fatty liver disease: practice Guideline by the American Association for the Study of Liver Diseases, American College of Gastroenterology, and the American Gastroenterological Association. Hepatology.

[46] Tucker-Drob EM, Reynolds CA, Finkel D, Pedersen NL (2014). Shared and unique genetic and environmental influences on aging-related changes in multiple cognitive abilities. Dev Psychol.

[47] Anstey KJ, Sargent-Cox K, Garde E, Cherbuin N, Butterworth P (2014). Cognitive development over 8 years in midlife and its association with cardiovascular risk factors. Neuropsychology.

[48] Singh-Manoux A, Sabia S, Lajnef M, Ferrie JE, Nabi H, Britton AR, Marmot MG, Shipley MJ (2008). History of coronary heart disease and cognitive performance in midlife: the Whitehall II study. Eur Heart J.

